# Identification of a Novel Functional Non-synonymous Single Nucleotide Polymorphism in Frizzled Class Receptor 6 Gene for Involvement in Depressive Symptoms

**DOI:** 10.3389/fnmol.2022.882396

**Published:** 2022-07-07

**Authors:** Haijun Han, Mengxiang Xu, Li Wen, Jiali Chen, Qiang Liu, Ju Wang, Ming D. Li, Zhongli Yang

**Affiliations:** ^1^State Key Laboratory for Diagnosis and Treatment of Infectious Diseases, National Clinical Research Center for Infectious Diseases, Collaborative Innovation Center for Diagnosis and Treatment of Infectious Diseases, The First Affiliated Hospital, Zhejiang University School of Medicine, Hangzhou, China; ^2^Department of Medical Engineering, Tianjin Medical University, Tianjin, China; ^3^Research Center for Air Pollution and Health, Zhejiang University, Hangzhou, China

**Keywords:** depression, genetic association, rs61753730, *Fzd6*, CRISPR/Cas9

## Abstract

Although numerous susceptibility loci for depression have been identified in recent years, their biological function and molecular mechanism remain largely unknown. By using an exome-wide association study for depressive symptoms assessed by the Center for Epidemiological Studies Depression (CES-D) score, we discovered a novel missense single nucleotide polymorphism (SNP), rs61753730 (Q152E), located in the fourth exon of the frizzled class receptor 6 gene (*FZD6*), which is a potential causal variant and is significantly associated with the CES-D score. Computer-based *in silico* analysis revealed that the protein configuration and stability, as well as the secondary structure of FZD6 differed greatly between the wild-type (WT) and Q152E mutant. We further found that rs61753730 significantly affected the luciferase activity and expression of *FZD6* in an allele-specific way. Finally, we generated *Fzd6*-knockin (*Fzd6*-KI) mice with rs61753730 mutation using the CRISPR/Cas9 genome editing system and found that these mice presented greater immobility in the forced swimming test, less preference for sucrose in the sucrose preference test, as well as decreased center entries, center time, and distance traveled in the open filed test compared with WT mice after exposed to chronic social defeat stress. These results indicate the involvement of rs61753730 in depression. Taken together, our findings demonstrate that SNP rs61753730 is a novel functional variant and plays an important role in depressive symptoms.

## Introduction

Major depressive disorder (MDD) has a high prevalence among psychiatric diseases (Charlson et al., 2019). It is not only a potentially fatal disease, with approximately 2% of affected patients committing suicide (Bostwick and Pankratz, 2000), but also one of the leading causes of lost productivity worldwide (Ustun et al., 2004; Ebmeier et al., 2006). It has been projected by the World Health Organization that MDD will become the cause of the greatest disease burden worldwide by 2030 (Malhi and Mann, 2018).

Major depressive disorder is moderately heritable with an estimated heritability of 37% (Sullivan et al., 2000; Kendler et al., 2006). Although a great number of susceptibility loci have been identified (Bosker et al., 2011; Wray et al., 2012, 2018; Consortium, 2015; Hyde et al., 2016; Direk et al., 2017; Power et al., 2017; Howard et al., 2019), most of them are synonymous variants and failed to be replicated in multiple independent studies. Even for a few of those identified non-synonymous variants, the biological functions and molecular mechanisms underlying their involvement in the pathogenesis of MDD are rarely studied (Kendall et al., 2021). Given the presence of high heterogeneity in depression, low chances of finding non-synonymous variants associated with MDD, and technical difficulties of characterizing those identified non-synonymous variants, the genetic and functional study of non-synonymous variants for MDD is challenging, as for other complex diseases (Ormel et al., 2019; Cai et al., 2020).

The Wnt/β-catenin pathway has been widely accepted to participate in the pathophysiology of mood disorders, including depression (Duman and Voleti, 2012; Sani et al., 2012). The frizzled (FZD) proteins are seven transmembrane-spanning receptors for Wnt ligands involved in this pathway (Huang and Klein, 2004). There is an increasing number of human and animal studies reporting that FZD family is related to depression and other psychiatric disorders. For example, Jang and colleagues reported that three SNPs in *FZD3* were significantly associated with early antidepressant responses in a clinical cohort (Jang et al., 2013). Calabro et al. (2020) found that SNP rs352428 located in *FZD3* was associated with depression and that G allele carriers showed a higher risk of previous depressive episode. *FZD3* was also reported to be highly associated with the risk of schizophrenia (Yang et al., 2003; Zhang et al., 2004). Liu et al. (2020) found that SNP rs10252923 in *FZD1* was significantly associated with schizophrenia. Voleti et al. (2012) reported *Fzd6* knockdown in rat brain resulted in depressive and anxiety behaviors. However, the genetic and mechanistical studies on FZD6 in depression are rare.

In the present study, we aimed not only to identify genetic variants associated significantly with depression but also to demonstrate their involvement at the molecular level. Specifically, we wanted to demonstrate, using various molecular techniques and behavioral tests in both *in vivo* and *in vitro* models, that non-synonymous variant rs61753730, located in the fourth exon of the *FZD6*, is a functional one and plays an important role in depression.

## Materials and Methods

### Subjects and Genotyping

The subjects used in this study were recruited during 2005–2011 primarily from the Mid-South states in the United States as part of the Mid-South Tobacco Case-Control (MSTCC) study. For a detailed description of these samples, please refer to our previously reported studies (Yang et al., 2015; Yang and Li, 2016). All materials related to subject recruitment were approved by the local ethics committees, and all subjects provided written informed consent.

Depressive symptoms were measured with a 20-item version of the Center for Epidemiological Studies Depression (CES-D) scale (Radloff, 1977), and the score of each subject from the baseline examination was used (Harlow et al., 1991; Kuchibhatla et al., 2012). Questionnaires assessing various other characteristics of interest were also administered, and individuals exhibiting other substance abuse (except smoking) or other psychiatric disorders (except depression) were excluded. As previously reported (Yang et al., 2015; Yang and Li, 2016; Jiang et al., 2019; Xu et al., 2020), genomic DNA was isolated from the peripheral blood of each patient, and genotyping was performed using the Illumina Infinium HumanExome BeadChip v 1.0 (Illumina Inc., San Diego, CA, United States). This exome chip includes more than 240,000 functional exonic variants that have been detected in several sequencing data sets, and it was designed to concentrate on rare variants.^1^ A total of 4,817 unrelated participants with both genotypic and phenotypic data was enrolled in this study. More detailed descriptions of these participants are shown in Supplementary Table 1.

### Exome-Wide Association Analysis

We excluded SNPs from further analysis if they: (1) were located on sex chromosomes or the mitochondrial genome; (2) had a call rate of <0.95; (3) deviated from the Hardy-Weinberg equilibrium at a *p*-value of <1 × 10^–6^; or (4) had a minor allele frequency (MAF) of <0.01. After these quality control steps, 38,943 SNPs and 4,817 individuals remained for the analyses reported in this paper.

Single-marker association analysis was carried out with a linear regression model adjusted for age, sex, ethnicity, smoking status, and the first three MDS using PLINK (Purcell et al., 2007). Association analysis results were considered significant after Bonferroni correction for the 38,943 SNPs (i.e., *p* < 0.05/38,943 = 1.28 × 10^–6^). Considering that only 11 SNPs were included on the Illumina Infinium HumanExome BeadChip for *FZD6*, we carried out SNP imputation with IMPUTE2 (Howie et al., 2012) after phasing in SHAPEIT2 (Delaneau et al., 2013) using the 1000 Genome Phase 3 data (Build 38) as a reference, which extended the number of SNPs in *FZD6* from 11 to 132 after excluding those with low imputation quality (info < 0.3). Then, we conducted gene-based association analysis to examine the presence of association between *FZD6* and the CES-D score using SNP-set Kernel Association Test (SKAT) software (Ionita-Laza et al., 2013). The EWAS dataset used in the current study has been submitted to public database and it is available from NCBI GEO (Accession: GSE148375).^2^

### *In silico* Analysis of Protein Variant Effect

SWISS-MODEL^3^ was used to generate a three-dimensional (3D) structure for wild-type (WT) FZD6 protein (Arnold et al., 2006). A template of human Smoothened in complex with cholesterol (SMTL ID 5I7d.1), which was searched through a BlastP algorithm against the PDB database, showed 27.18% identity and was used for homology modeling of FZD6. The mutant models also were constructed and analyzed in relation to the WT by PyMOL.^4^

Changes in protein stability resulting from a single point mutation were predicted by I-Mutant 3.0^5^ based on the Support Vector Machine (SVM) algorithm. It predicted the ΔΔG value as a regression estimator and sign of the stability change, and classified mutations into three categories: neutral (−0.5 − 0.5), decrease (<−0.5), and increase (>0.5) (Schymkowitz et al., 2005). We also used Mutation Cutoff Scanning Matrix (mCSM; Pires et al., 2014b), which applies the concept of graph-based structural signatures to predict the effect of a single point mutation on protein stability. DUET, an integrated computational web server, applies mCSM to predict missense mutation effects on protein stability (Pires et al., 2014a). The ΔΔG value < 0 means a decrease in the stability; otherwise, an increase in the stability. NetSurfP 2.0^6^ is a sequence-based tool to predict protein structures, including secondary structure, structural disorder, and backbone dihedral angles for each residue of the input sequences (Klausen et al., 2019).

### Cell Culture

Human embryonic kidney 293T (HEK293T) cells were purchased from the American Type Culture Collection (Manassas, VA, United States). Cells were cultured in Dulbecco’s Modified Eagle Medium (Hyclone, Logan, UT, United States) supplemented with 10% fetal bovine serum (Gibco, Grand Island, NY, United States) and 1% penicillin/streptomycin (Gibco) in a humidified atmosphere of 5% CO_2_ at 37°C.

### Luciferase and mRNA Decay Assays

The *FZD6* DNA fragment containing the C allele of rs61753730 was synthesized and cloned into the pGL3-promoter (Promega, Madison, WI, United States). The G allele was introduced into the rs61753730-C allele construct using a QuikChange Lightning Multi Site-Directed Mutagenesis Kit (Agilent, Santa Clara, CA, United States), and its sequence was verified by Sanger sequencing. When HEK293T cells reached 80–90% confluence, transfection was conducted with a pGL3-promoter construct and pRL-TK vector (Promega) by Lipofectamine 2000.

For the luciferase assay, cells were lysed 48 h after transfection and assayed with the Dual-Luciferase Report Assay System (Promega). For the mRNA decay assay, actinomycin D (5 μg/ml) was added 24 h after transfection, and total RNA was extracted at 0, 1, or 3 h. The quantitative real-time PCR (qRT-PCR) was then conducted to determine the relative expression of the luciferase reporter gene.

### RNA Extraction and Quantitative Real-Time PCR

Total RNA was isolated from the cells after treatment and homogenized by an ultrasonic disruptor in TRIzol reagent (Life Technologies, Grand Island, NY, United States) according to the manufacturer’s instructions. The purity and quantity of total RNA were measured at the optical densities of 260 and 280 nm with NanoDrop 2000 (Thermo Scientific, Waltham, MA, United States). First-strand cDNAs were synthesized from 1 μg of total RNA using an iScript cDNA Synthesis Kit (Bio-Rad, Hercules, CA, United States). The qRT-PCR amplification was conducted in a volume of 10 μl containing 5 μl of 2 × Power SYBR Green PCR Master Mix (Applied Biosystems), mixed with equal amounts of sense and antisense primers (2.5 μl; final concentration 250 nM each), and 2.5 μl of diluted cDNA in a 384-well plate using 7900 HT Fast Real Time PCR system (Applied Biosystems) as described previously (Yang et al., 2016; Han et al., 2019; Chen et al., 2020; Fan et al., 2021). The PCR conditions were as follows: 50°C for 2 min, 95°C for 10 min, followed by 40 cycles of 95°C for 15 s and 60°C for 1 min. The primers for the reporter gene are forward: 5′-TGCACATATCGAGGTGGACATC-3′ and reverse: 5′-TGCCAACCGAACGGACAT-3′; both were synthesized at Sango Biotech (Shanghai) Co., Ltd. The expression of the gene of interest was normalized to the housekeeping gene glyceraldehyde 3-phosphate dehydrogenase (*GAPDH*) and then analyzed using a comparative *C*_*t*_ method (Winer et al., 1999).

### Animals

All animals were purchased from GemPharmatech Co., Ltd., (Nanjing, China) and maintained in the Animal Core Facility at Zhejiang University. Mice were kept under the standard housing conditions on a 12-h light/dark cycle. Food and water were freely available. Mice were deeply anesthetized before sacrifice. All experimental procedures used in the study were approved by the Animal Use and Care Committee of the First Affiliated Hospital of Zhejiang University.

### Generation of *Fzd6*-Knockin Mice Using CRISPR/Cas9 Editing System

The *Fzd6*-knockin (*Fzd6*-KI) mice based on C57BL/6J were generated *via* the optimized CRISPR/Cas9 system at GemPharmatech Co., Ltd., with almost no off-target effects detected (Shen et al., 2014). Briefly, Cas9 mRNA, sgRNA, and donor compounds were co-microinjected into fertilized C57BL/6J mouse zygotes. The sgRNA directed to Cas9 endonuclease cleavage near the mutation site and created a double-strand break. Such a break was repaired and resulted in a Q152E point mutation, which was then inserted into exon 4 by homologous recombination and we obtained the *Fzd6*-KI mice.

DNA was isolated from the tail of each pup, and PCR was conducted for genotyping. The PCR products were electrophorized to screen if it is wild-type or mutant mouse. Then, Sanger sequencing was used to validate the gene sequence, especially at the mutant site. The sequence of sgRNA was 5′-CACCAAAATCCAATGTCTCT-3′, and the donor sequence was 5′-CCTGTAAC TTCTCATCCACACACAGAGCTTTCTGGGCCACAGAAGA AATCAGATGAAGTCCCACGCGACATTGGATTTTGGTGT CCAAAGCACCTTAGGACTTCCGGGGACCAAGGCTAT-3′. The primers for genotyping were forward 1: 5′- TCT GTGAATGCAGCAAAGTCATGG-3′, reverse 1: 5′- ATCCA ATGTCGCGTGGGACTTC-3′; forward 2: 5′-TCTGTGAATGC AGCAAAGTCATGG-3′, reverse 2: 5′-GTCTCTCTGGGTATC TGAATCGTC-3′. The primers for sequencing was 5′-GTC TCTCTGGGTATCTGAATCGTC-3′. After genotyping, through crossbreeding of the heterozygote mice, we obtained *Fzd6*-KI homozygous and WT mice, which were used for the following studies. For the identification of some representative animals with different genotypes, please see the attached Supplementary Material.

### Behavioral Tests

The adult (2–3-month old) male and female homozygous *Fzd6*-KI and WT mice were subjected to behavioral tests (*n* = 7–9 mice/genotype). All mice were habituated to the testing room for 1 h prior to each test. After completion of all relevant behavioral tests, all mice were sacrificed.

### Chronic Social Defeat Stress Model for Depression

The chronic social defeat stress (CSDS) model of depression was carried out as described previously (Golden et al., 2011; Harris et al., 2018; Shen et al., 2019). Briefly, prior to each experiment, more than the number of 50 retired male breeder CD1 mice (known as aggressor mice) were screened for three consecutive days to determine the aggressive characteristics of each animal, which was done by placing the screener C57BL/6J mouse directly into the home cage of the CD1 mice for 3 min with the presence of an aggressor. The following two criteria were used to select the CD1 aggressor mice: (1) mouse must attack in at least two consecutive sessions during three 3-min screening sessions; and (2) the latency to initial aggression must be less than 60 s. Then, each experimental mouse (male *Fzd6*-KI mouse) was introduced into the home cage of a CD1 aggressor for 5 min. At the end of 5 min of social defeat, the experimental mice were moved to the other side separated from the aggressors with a perforated Plexiglas divider where animals were maintained for 24 h. After 24 h of sensory but not physical contact, the above procedure was repeated daily for 10 consecutive days. The non-defeated control animals were also pair-housed with an identical home cage setup. The two mice on each side were never allowed physical or sensory contact with CD1 mice or their home mates. Each experimental mouse was faced with a different aggressor every day. The social interaction test was conducted 24 h later after 10 days of social defeat.

### Social Interaction Test

Social avoidance behavior was evaluated by the social interaction (SI) test in an open-field apparatus (44 cm × 44 cm × 44 cm) consisting of two consecutive phases each for 2.5 min. During the first phase (target absent), experimental animals were placed individually in the apparatus containing an empty wire-mesh enclosure (10 cm × 6.5 cm × 25 cm) with the target CD1 aggressor absent, and their movements were monitored with Any-maze video tracking software. In the second phase (target present), a novel aggressive CD1 mouse was placed in the wire-mesh enclosure with the same metrics measured. Between the two phases, the experimental animals were returned to their home cages for an approximately 30 s interval. The time spent in the interaction zone (a 14 cm × 26 cm rectangular area projecting 8 cm around the wire-mesh enclosure) and in the corner zones (a 9 cm × 9 cm area projecting from both corner joints opposing the wire-mesh enclosure) were counted.

### Open Field Test

The experimental animals were placed individually in the center of the open field test (OFT) apparatus (44 cm × 44 cm × 44 cm) with a dim light for 10 min. A video camera was arranged directly above the apparatus to monitor the movement of each animal. Locomotor activity was measured as the total distance traveled. Anxiety-like behavior was defined by the distance traveled, time spent in the central zone (15 cm × 15 cm), and center entries during the 10 min period. The apparatus was cleaned with 75% ethanol after each experiment. Data were collected by Any-maze software (Any-maze, Stoelting, Wood Dale, IL, United States).

### Forced Swim Test

The forced swim test (FST) was conducted as previously described (Yang et al., 2018). Each experimental animal was placed individually in a cylinder (12 cm diameter; 25 cm height) filled with water (23–25°C) and allowed to swim for 6 min. The depth of water in the cylinder (∼16 cm) was set to prevent animals from touching the bottom with their tails or hind limbs. The behaviors were recorded with a camera from the side. The total duration of immobility (no movement except keeping the head above water) in the last 4 min was counted.

### Sucrose Preference Test

The sucrose preference test (SPT) was conducted according to published protocols (Monteggia et al., 2007; Chaudhury et al., 2013; Shen et al., 2019) with minor changes. Animals were single housed and habituated with two bottles of water for two consecutive days. After habituation, the mice were allowed free access to a two-bottle choice of 2% sucrose solution or tap water. The positions of the two bottles were switched (left to right and right to left) every 6 h to avoid side preference. The total duration of the test was 48 h. The bottles were weighed at the start and end of each testing period. Sucrose preference was calculated as sucrose consumption/(water consumption + sucrose consumption) × 100% during the test phase.

### Statistical Analyses

All experimental data were analyzed with GraphPad Prism 8.0 (San Diego, CA, United States). All data are presented as means ± standard error of the mean (SEM). We used a two-tailed unpaired *t*-test for two-group comparisons and one-way ANOVA followed by Bonferroni multiple testing for more than two-group comparisons. Two-way ANOVA was applied to compare data from the behavioral tests after CSDS with two factors (Genotype × Stress). A *p*-value of <0.05 was considered statistically significant.

## Results

### Characteristics of Samples in This Study

The characteristics of the study population are presented in Supplementary Table 1. A total of 4,817 subjects with an average age of 42.8 years (± 13.5; SD) were included. Of them, 3,279 (68.1%) were self-reported African Americans, and 45.2% were males. In line with the population-based design of the study, the mean depressive symptom scores were 6.3 (± 9.5; SD) for the 20-item version of the CES-D.

### Single-Marker Based Association Analysis

**FIGURE 1:**
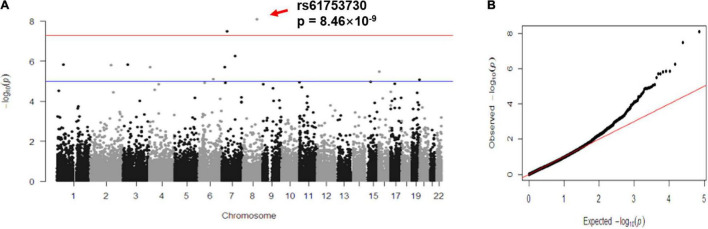
Genetic findings of rs61753730 in *FZD6* associated with depressive symptoms. **(A)** Manhattan plot of all results from exome-wide association study (EWAS) for CES-D score according to chromosome location. **(B)** Quantile–quantile plot of observed vs. expected –log_10_ (*p*-value) of SNPs from EWAS. λ, genomic inflation factor, estimated at close to 1. Red arrow indicates the location of rs61753730.

Table 1 lists the association results of the top 11 SNPs with *p* < 1 × 10^–5^, among which two loci showed significant association with the CES-D score after Bonferroni correction: rs61753730 (Chr. 8: 104336788; MAF = 0.012; *p* = 8.46 × 10^–9^) in *FZD6*; and rs35024632 (Chr. 7: 38433726; MAF = 0.025; *p* = 3.08 × 10^–8^) in the amphiphysin (*AMPH*) gene. Figure 1A shows the Manhattan plot of the association results by chromosome location, and two SNPs, located in chromosomes 7 and 8, showed signal above the commonly defined genome-wide significance threshold (*p* < 5 × 10^–8^) (Sullivan et al., 2009). Of these two SNPs, both are missense variants resulting in amino acid changes in their encoded proteins (rs61753730 from glutamine to glutamate; rs35024632 from lysine to threonine). Figure 1B depicts the quantile-quantile plot of observed vs. expected –log_10_ (*p*- value) for all 38,943 SNPs. The distribution of *p*- values was similar to what would be expected by chance, with a genomic control λ value of 1.000, indicating no detectable inflation of test statistics (Devlin and Roeder, 1999).

**TABLE 1:** A list of top 11 single nucleotide polymorphisms (SNPs) with *p* < 1 × 10^–5^ resulted from genome-wide association analysis.

db SNP ID	Chr.	Position	Gene	Alleles	MAF	Function	Amino acid change	*P*-value
rs61753730	8	104336788	*FZD6*	[C/G]	0.01214	Missense	Gln120Glu/Gln152Glu	**8.46E**−**09**
rs35024632	7	38433726	*AMPH*	[T/G]	0.02545	Missense	Lys454Thr/Lys496Thr	**3.08E**−**08**
rs117406702	7	100346094	*ZAN*	[A/G]	0.01443	Splice	NA	5.46E−07
rs62620991	1	46669276	*LURAP1*	[A/C]	0.01121	Missense	Met60Leu	1.41E−06
rs74424604	3	36931397	*TRANK1*	[T/C]	0.016	Missense	Arg233His	1.42E−06
rs6710212	2	152490458	*NEB*	[A/G]	0.0518	Missense	Cys3042Arg	1.56E−06
rs72658825	7	21934511	*DNAH11*	[A/G]	0.01766	Missense	Ala4322Thr	1.95E−06
rs61740788	4	4440183	*STX18*	[T/G]	0.0503	Missense	Glu184Ala	1.97E−06
rs61731432	16	3254418	*OR1F1*	[G/C]	0.02181	Missense	Pro58Ala	3.44E−06
rs61738623	6	109468136	*CEP57L1*	[A/G]	0.01195	Synonymous	Lys112Lys	8.21E−06
rs114567837	19	55870379	*FAM71E2*	[A/C]	0.02928	Missense	Glu619Asp	8.40E−06

*All SNP shown in the table are ranked based on the order of p-value. Three significantly associated SNPs after Bonferroni correction (p < 1.28 × 10^–6^) are given in bold. Chr., chromosome; MAF, minor allele frequencies; NA, not applicable.*

To characterize the most significantly associated SNP rs61753730 in *FZD6*, we performed gene-based association analysis for 11 genotyped and 121 imputed SNPs (info > 0.3) located in the gene as well as in a 2-kb region both upstream and downstream, which showed a significant association of *FZD6* with the CES-D score (*p* = 0.0078). Of these SNPs, 102 were rare and 30 were common variants.

### Allele-Specific Effect of rs61753730 on Frizzled Class Receptor 6 Gene

**FIGURE 2:**
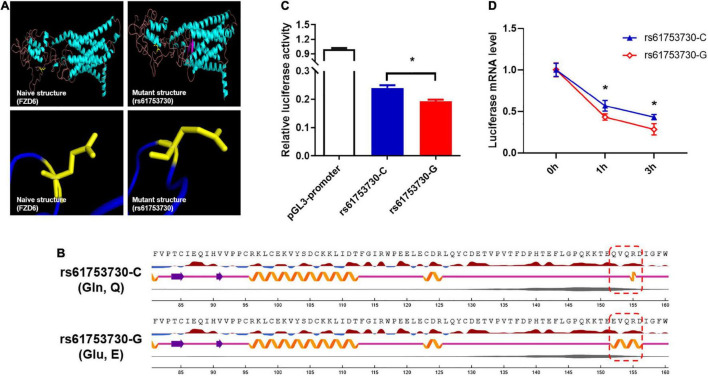
Allele-specific effects of rs61753730 on *FZD6*. **(A)** Effect of rs61753730 mutation on predicted structure of FZD6 protein generated by PyMOL. Upper panel: Naive 3D structure of FZD6 and mutant structure of rs61753730 (Q152E). Bottom panel: Naive structure of FZD6 shows glutamine at position 152, and predicted mutant structure of rs61753730 shows glutamic acid at corresponding position. **(B)** Effect of rs61753730 mutation on secondary structure of FZD6 at position 152 predicted by NetSurfP-2.0. Box with dashed line in red indicates different structure of WT (C) and mutant SNP (G). **(C)** Effect of rs61753730 on luciferase activity. HEK293T cells were transfected with rs61753730 C- and G-allele constructs of pGL3-promoter vector. Luciferase activity was measured 48 h later. Data are reported as relative firefly luciferase activity normalized to *Renilla* luciferase activity and represented as mean ± SEM (*n* = 3). *p < 0.05. **(D)** Effect of rs61753730 on mRNA stability of luciferase reporter gene. HEK293T cells were transfected with rs61753730 C- and G-allele constructs of pGL3-promoter vector for 24 h prior to adding actinomycin D (5 μg/ml). The mRNA of luciferase expression was measured at different times (0, 1, and 3 h) and normalized to *GAPDH*. Data are presented as mean ± SEM (*n* = 3). *p < 0.05.

To further investigate the biological role of rs61753730 on FZD6 in depression, we carried out *in silico* analysis and found that the different alleles of rs61753730 lead to an altered protein configuration (Figure 2A). Although no obvious difference was observed in the physiochemical characteristics of the protein and disulfide bonds, several distinctions were discovered in the secondary structure and transmembrane helices (Supplementary Table 2). On the basis of the prediction by NetSurfP-2.0, the secondary structure was quite different at position 152 of FZD6 (Figure 2B), which was a coil in the WT (Gln, Q) but a helix in the mutant FZD6 (Glu, E). We further predicted the stability of the protein after mutation by I-Mutant 3.0 and found that the ΔΔG value was −0.18 kcal/mol, which means the mutation decreased the stability of the FZD6 protein because of the amino acid change from Gln (rs61753730-C) to Glu (rs61753730-G) at position 152. These findings were further supported by another structure-based prediction tool mCSM with an online server DUET (ΔΔG = −0.039 kcal/mol). Together, these *in silico* results indicated that the different allele of rs61753730 changed the structure of FZD6.

In determining the allele-specific function of rs61753730 on *FZD6* experimentally, HEK293T cells transfected with the rs61753730-G allele construct showed a 20% decrease in luciferase activity (*p* = 0.014) compared with the cells transfected with the rs61753730-C allele (Figure 2C). Further, when the cells were treated with actinomycin D, the mRNA quantity of the luciferase reporter gene was significantly reduced in both C and G allele-transfected cells, whereas the construct carrying the rs61753730-C allele decayed significantly more slowly than that containing the G allele, with a *p*-value of 0.0349 and 0.0267 after 1 and 3 h, respectively, of actinomycin D treatment (Figure 2D).

By analyzing the expression quantitative trait loci (eQTL) dataset from GTEx Portal, we found that rs61753730 showed eQTL regulation with allele-specific significance on *FZD6* mRNA expression, especially in the cerebellum (*p* = 1.3 × 10^–5^), a brain region implicated in depression (Depping et al., 2018). These results demonstrated that the missense mutation rs61753730 contributed to the effect of FZD6 on depression.

### Generation of *Fzd6*-Knockin Mice

As shown in Supplementary Figures 1A,B, the glutamine residue at position 152 (rs61753730-C) was highly conserved from human to mouse and rat, suggesting it has functional importance, and the mouse model could be used to investigate the biological role of rs61753730 in depressive symptoms. Supplementary Figure 1C shows the strategy used to generate the rs61753730 mutant *Fzd6*-KI mice with the CRISPR/Cas9 system. Since rs61753730 is located in the fourth exon of FZD6, the CRISPR/Cas9 system targeted to it to obtain the *Fzd6*-KI mice carrying G allele. The sequence was validated by Sanger sequencing with the C allele in WT mice and the G allele in *Fzd6*-KI mice (Supplementary Figure 1D).

### Construction of Chronic Social Defeat Stress Depression Model in *Fzd6*-Knockin Mice

**FIGURE 3:**
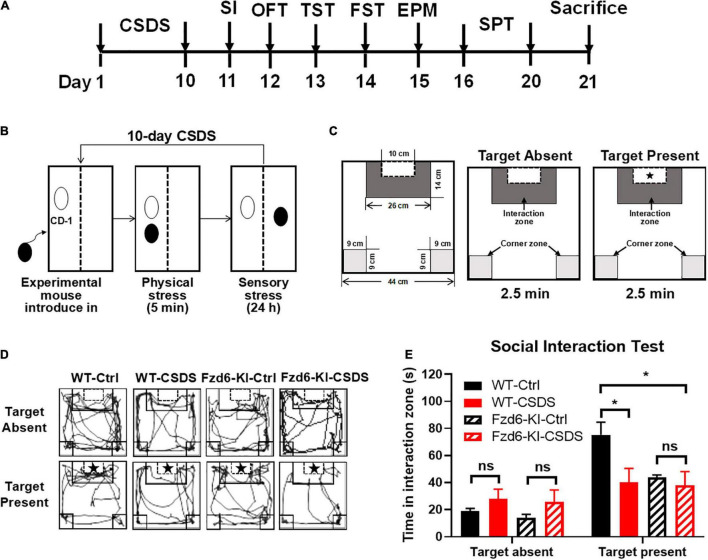
Construction of 10-day CSDS depressive model in *Fzd6*-knockin (*Fzd6*-KI) mice. **(A)** Schematic diagram of experimental design. **(B)** Schematic of 10-day CSDS model. **(C)** Schematic of open field arena and zones for social interaction (SI) test in absence or presence of target CD-1 mice. Pentagram marks target mice. **(D)** Representative tracking plot of SI test in absence (top line) and presence (bottom line) of target. **(E)** Time spent in interaction zone in absence and presence of target in both wild-type and *Fzd6*-KI mice after CSDS. *p < 0.05, ns, not significant.

To characterize the role of rs61753730 in depression in the mouse model, the 10-day CSDS-induced depression model, a well-validated and widely used model (Golden et al., 2011), was employed (Figures 3A,B). After the 10-day CSDS, the social interaction test was performed (Figures 3C–E). When there was no target, the two-way ANOVA showed no significant interaction for Stress × Genotype (*F*_(1,13)_ = 0.04783, *p* = 0.8303), and both the Genotype factor (*F*_(1,13)_ = 0.3138, *p* = 0.5849) and Stress factor (*F*_(1,13)_ = 2.643, *p* = 0.1280) also showed no significant difference, respectively, which means the baseline is similar for the four groups. When the target present, although the results showed no significant interactions for Stress × Genotype (*F*_(1,13)_ = 2.212, *p* = 0.1608) and the Genotype factor (*F*_(1,13)_ = 2.212, *p* = 0.1608), the stress factor showed a close to significant difference (*F*_(1,13)_ = 4.353, *p* = 0.0572). As shown in Figure 3E, in the presence of target mice, the WT mice who subjected to CSDS spent less time in the interaction zone than the WT mice without CSDS (*p* < 0.05), and the *Fzd6*-KI mice spent less time in the interaction zone compared with the WT mice (*p* < 0.05). No significant difference was observed between *Fzd6*-KI mice whether subjected to CSDS or not (*p* > 0.05). It suggests that the *Fzd6*-KI mice is not susceptible to stress, however, this type of mice still can present the similar depressive-like symptoms similar with WT-CSDS mice. Thus, more depressive-like and anxiety-like behaviors need to be conducted to verify this phenomenon.

### Alterations of rs61753730 Mutation on Depressive-Like and Anxiety-Like Behaviors After Chronic Social Defeat Stress in *Fzd6*-Knockin Mice

**FIGURE 4:**
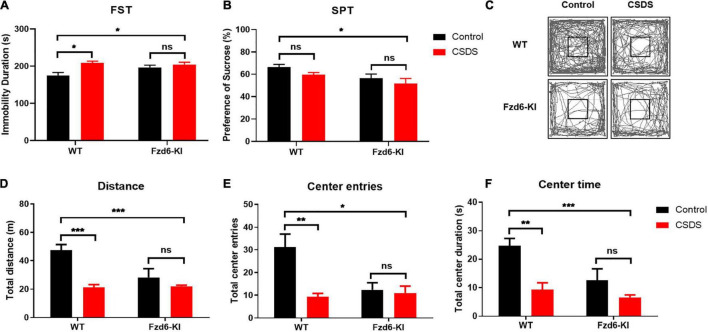
Changes of depressive- and anxiety-like behaviors in *Fzd6*-KI mice after CSDS. **(A,B)** Depressive-like behaviors. Graphs show immobility time, scored in seconds, in panel **(A)** forced swimming test (FST) and percentage of sucrose consumption over total fluid consumption in panel **(B)** sucrose preference test (SPT). **(C–F)** Anxiety-like behaviors. **(C)** Representative tracking plot of open field test (OFT). Graphs present **(D)** total distance traveled, **(E)** center entries, and **(F)** time spent in center area. Animals were tested in 3–5 mice/group. Data were analyzed using two-way ANOVA, and all data are presented as mean ± SEM. *p < 0.05, **p < 0.01, ***p < 0.001, ns, not significant.

To identify the depressive-like behaviors in *Fzd6*-KI mice, we carried out FST and SPT experiments and used the measurement of immobility duration and sucrose preference ratio to evaluate anhedonia. In the FST, two-way ANOVA showed significance for Stress factor (*F*_(1,13)_ = 9.415, *p* = 0.0090), but not for Genotype factor (*F*_(1,13)_ = 1.631, *p* = 0.2239) and for their interactions (*F*_(1,13)_ = 3.312, *p* = 0.0919). Compared with the WT control mice, the immobility duration increased significantly (*p* < 0.05; Figure 4A) after the WT mice were subjected to CSDS. The *Fzd6*-KI mice exposed to CSDS also showed significantly greater immobility compared with the WT control mice (*p* < 0.05), whereas no significant difference was observed between the *Fzd6*-KI mice with and without CSDS exposure. In the SPT, two-way ANOVA showed that significant difference for Genotype factor (*F*_(1,12)_ = 7.452, *p* = 0.0183), but not for the Stress factor (*F*_(1,12)_ = 3.088, *p* = 0.1044) and their interactions (*F*_(1,12)_ = 0.08952, *p* = 0.7699). Although no significant difference in the sucrose preference ratio was detected between the WT mice exposed and not exposed to CSDS (Figure 4B), the results showed the same trend; and *Fzd6*-KI mice exposed to CSDS displayed significantly less sucrose preference than the WT control mice (*p* < 0.05).

As anxiety-like behaviors correlated highly with depression, we measured these behaviors in *Fzd6*-KI mice with the OFT, which was measured by the number of times the mice entered or spent time in the central area. Two-way ANOVA showed significant differences for Stress × Genotype interactions for total distance traveled (*F*_(1,13)_ = 8.376, *p* = 0.0125) and center entries (*F*_(1,13)_ = 6.317, *p* = 0.0259), but not for time spent in the center zone (*F*_(1,13)_ = 3.289, *p* = 0.0929). As shown in Figure 4C, the locomotor activities with total distance traveled (Figure 4D), center entries (Figure 4E), and time spent in the center zone (Figure 4F) were all greatly decreased after the WT mice were subjected to CSDS (*p* < 0.01), and the *Fzd6*-KI mice with or without CSDS exposure showed a comparable reduction. In *Fzd6*-KI mice exposed to CSDS, it also presented a significant decrease in locomotor activities compared with the WT control mice (*p* < 0.05), but there was no significant difference in *Fzd6*-KI mice with or without CSDS exposure. Taken together, these behavioral results strongly indicated that the *Fzd6*-KI mice presented depressive symptoms like those in WT mice subjected to CSDS, suggesting an important role of rs61753730 in depression.

## Discussion

In this study, we found two SNPs (*P*_*rs*61753730_ = 8.46 × 10^–9^; *P*_*rs*35024632_ = 3.08 × 10^–8^) to be significantly associated with the CES-D score at a genome-wide significance level. We further revealed rs61753730 to be a functional SNP modulating the structure and function of FZD6 in an allele-specific manner. Finally, we generated the single point mutation *Fzd6*-KI mouse model, which expressed depressive-like behaviors. All these findings strongly demonstrated the functional roles of rs61753730 in depression.

In recent years, numerous studies have been conducted to identify susceptible SNPs for depression, and most identified variants found so far appear to be non-coding (Wray et al., 2018; Howard et al., 2019). For example, Wray et al. (2018) found 44 significant loci associated with depression, and all of them were non-coding. In another study, Howard et al. (2019) identified 102 variants associated with depression, of which only one SNP was coding, this one located in the exon region of the DENN domain containing 1B (*DENND1B*). As we all know, those SNPs that alter the amino acid coding (i.e., a non-synonymous SNP) are likely to play more important roles in a disease or phenotype of interest because the missense SNP can change the protein structure and thus alter the function of the protein (Ormel et al., 2019). Luckily, our current study discovered a missense SNP rs61753730 in *FZD6* that is significantly associated with depression, in which the person who carries rs61753730-G (the risk allele) may have an increased risk for depression.

The FZD proteins are Wnt ligands in Wnt/β-catenin pathway (Huang and Klein, 2004). A number of studies have reported that the member in FZD family is involved in depression or antidepressant. Jang et al. (2013) identified FZD3 as a molecular target for antidepressant treatments in both animal model and human studies. Recently, Li et al. (2021) found that FZD7 represents a novel GPCR regulator in depression, which is dysregulated in chronic unpredictable mild stress (CUMS) mouse model. This gene was also identified as tentative biomarkers for antidepressant drug resistance through *in vitro* study (Breitfeld et al., 2017). Okamoto et al. (2010) indicated that antidepressants can upregulate *Fzd9* expression in the hippocampus of rat. Furthermore, FZD6 is not only implicated in the process of human cancer (Corda and Sala, 2017), neural tube defects (De Marco et al., 2012; Shi et al., 2014), and brain morphogenesis defect (Stuebner et al., 2010), but also in depression. A previously reported animal study demonstrated that *Fzd6* knockdown in the hippocampus of rats induces depressive-like and anxiety-like behaviors (Voleti et al., 2012). Knocking down of Wnt2/Wnt3, the ligand of FZD6, also results in depressive-like behaviors; in contrast, overexpression of Wnt2/Wnt3 reverses depressive-like behaviors (Zhou et al., 2016). These studies highlight the essential role of FZD6 in depression.

The change in an amino acid to create the missense SNP may cause unstable structure and dysfunction of the gene where it occurs. To define the biological role of SNP rs61753730 in the regulation of FZD6 in depression, we carried out a series of investigations at different levels. The computer-based *in silico* technique is an effective way to predict the potential function of an SNP of interest prior to initiating experiments in a “wet” laboratory (Rajasekaran et al., 2007; Khalid and Almaghrabi, 2020). Through predictions by various software programs, we found the protein configuration and stability of FZD6 to be altered by the change of glutamine to glutamic acid at position 152. Although most of the risk variants that regulate gene expression are located in non-coding regions, the missense mutations also exerted regulatory functions as reflected in luciferase activity (Shimajiri et al., 2001; Kato et al., 2003; van Steensel et al., 2009; Hemming et al., 2020). Our functional studies demonstrated that the rs61753730-G allele significantly decreased the activity and mRNA stability of the luciferase reporter gene. The eQTL analysis can be used to link the risk SNP to the expression of a specific gene or nearby genes (Fu et al., 2012). Through the GTEx Portal database, we found rs61753730 to be an eQTL regulating *FZD6* mRNA expression in an allele-specific manner. All these results imply that rs61753730 is a functional SNP modulating FZD6.

Animals with depression usually exhibited anhedonia, anxiety, or despair-like behaviors (Yan et al., 2010; Wang et al., 2017; Hao et al., 2019). The behavioral paradigms such as TST or FST can be used to assess despairing behavior. The anxiety-like behavior can be determined by OFT or EPM, and the anhedonia can be detected by SPT (Yan et al., 2010; Wang et al., 2017; Hao et al., 2019). In this study, to better determine the functional role of rs61753730 in depression, we generated an *Fzd6*-KI mouse model with rs61753730 mutation by applying the cutting-edge CRISPR/Cas9 technique, which can target even a single nucleotide (Heidenreich and Zhang, 2016; Pickar-Oliver and Gersbach, 2019; Wu et al., 2019, 2020). By performing various animal behavior tests, we found the *Fzd6*-KI mice presented a significant increase in immobility in FST, reduced sucrose preference, and decreased locomotor activities in OFT after exposure to 10-day CSDS, suggesting these animals presented anhedonia, anxiety, or despair. Consistent with our behavioral results, a previous study has shown that partial *Fzd6* knockdown in the brain of rats induced anhedonic responses and anxiety, such as decreased sucrose preference, suppressed feeding, and altered performance in the EPM (Voleti et al., 2012). In a most recent paper, Morel et al. (2022) utilized the same paradigm CSDS to induce anxiety- and depressive-like phenotypes, and the results showed that the ventral tegmental area (VTA) to basolateral amygdala (BLA) dopamine projection is an important circuit mechanism for anxiety-like behaviors and anxiety-depressive comorbid conditions, but not for depressive-like behaviors in mice. Thus, the behavioral alterations observed in the present study further demonstrated the functional role of rs61753730 in depression, which may attribute to a VTA→BLA dopamine neural circuit.

In summary, we carried out an exome-wide association study of the CES-D score with 4,817 samples and identified two significant SNPs at the genome-wide level. The strongest signal was rs61753730, a missense variant predicted to alter protein configuration and decrease the structural stability of FZD6 in an allele-specific manner. Through animal behavioral tests in chronic stress, we further showed that rs61753730 in *FZD6* is highly related to depressive symptoms. Taken together, our current results systematically elucidated that rs61753730 is a functional SNP playing significant roles in depression.

## Data Availability Statement

The raw data supporting the conclusions of this article will be made available by the authors, without undue reservation.

## Ethics Statement

The studies involving human participants were reviewed and approved by the Institutional Review Boards of the University of Virginia and University of Mississippi Medical Center. The patients/participants provided their written informed consent to participate in this study. The animal study was reviewed and approved by Animal Use and Care Committee of The First Affiliated Hospital of Zhejiang University.

## Author Contributions

HH, MX, LW, and JC carried out the molecular experiments. LW, QL, and JW performed the bioinformatic analyses. HH, LW, MDL, and ZY wrote and edited the manuscript. MDL and ZY conceptualized and supervised the project. All authors contributed to the article and approved the submitted version.

## Conflict of Interest

The authors declare that the research was conducted in the absence of any commercial or financial relationships that could be construed as a potential conflict of interest.

## Publisher’s Note

All claims expressed in this article are solely those of the authors and do not necessarily represent those of their affiliated organizations, or those of the publisher, the editors and the reviewers. Any product that may be evaluated in this article, or claim that may be made by its manufacturer, is not guaranteed or endorsed by the publisher.

## Footnotes

1
https://genome.sph.umich.edu/wiki/Exome_Chip_Design

2
https://www.ncbi.nlm.nih.gov/geo/

3
http://swissmodel.expasy.org/interactive

4
http://pymol.org/

5
http://gpcr2.biocomp.unibo.it/cgi/predictors/I-Mutant3.0/I-Mutant3.0.cgi

6
https://services.healthtech.dtu.dk/service.php?NetSurfP-2.0

## Supplementary Material

Supplementary Figure 1 Generation of *Fzd6-KI* mice by CRISPR/Cas9 system. **(A,B)** Conservation analysis of rs61753730 (Q152E). **(A)** The UCSC Genome Browser Alignments depict nucleotide alignment of human *FZD6* gene and homologs from other species. Position of rs61753730 variant is indicated by dashed red line. **(B)** Glutamine (Q) is conserved at same point in human, mouse, rat, and other animals. **(C)** Schematic of strategy for construction of *Fzd6* point-mutation mice *via* the CRISPR/Cas9 system. The rs61753730 is located in fourth exon of *Fzd6*. Red asterisk indicates mutation site. **(D)** Sequencing chromatogram to identify WT (with C allele) and *Fzd6*-KI (with G allele) mice from tail genome DNA. Dashed red line marks location of rs61753730 in two types of mice.
