# Supplementary material for: Identification of a Novel Functional Non-synonymous Single Nucleotide Polymorphism in Frizzled Class Receptor 6 Gene for Involvement in Depressive Symptoms
Source: Front Mol Neurosci. 2022 Jul 7;15:882396. doi: 10.3389/fnmol.2022.882396 (PMC9302575; doi:10.3389/fnmol.2022.882396)
Supplement: Supplementary file 1 [file Data_Sheet_1.DOCX]

**Table S1.** Descriptive characteristics of samples used in the study

| **Characteristic** | **Statistics** |
| --- | --- |
| Sample Size | 4,817 |
| African Americans (%) | 68.1% |
| Age (mean ± SD; years) | 42.8 ± 13.5 |
| Males (%) | 45.2% |
| Smoking rate (%) | 48.9% |
| CES-D score (mean ± SD) | 6.3 ± 9.5 |

Notes: SD = standard deviation; CES-D = Center for Epidemiological Studies Depression.

**Table S2.** Effect of SNP rs61753730 on the physiochemical properties and secondary structure of FZD6 protein

| **Software** | **Item** | **rs61753730 (C>G)** | |
| --- | --- | --- | --- |
|  |  | **C (Gln)** | **G (Glu)** |
| ProtParam | No. of amino acids | 706 | 706 |
|  | Molecular weight | 79292.05 | 79293.04 |
|  | Theoretical pI | 8.29 | 8.21 |
|  | Atomic composition | C_3576_H_5564_N_934_O_1007_S_48_ | C_3576_H_5563_N_933_O_1008_S_48_ |
|  | Instability index | 45.79 | 46.08 |
|  | Aliphatic index | 85.86 | 85.86 |
|  | Grand average of hydropathicity | 0.001 | 0.001 |
| Scratch | Disulfide bonds | 12 | 12 |
| SOPMA | Alpha helix | 36.12% (255) | 36.26% (256) |
|  | Beta turn | 7.51% (53) | 7.51% (53) |
|  | Extended strand | 18.13% (128) | 17.99% (127) |
|  | Random coil | 38.24% (270) | 38.24% (270) |
| PredictProtein | Transmembrane helices | 1: 207-223 | 1: 208-222 |
|  |  | 2: 235-254 | 2: 238-255 |
|  |  | 3: 284-304 | 3: 283-302 |
|  |  | 4: 324-345 | 4: 325-345 |
|  |  | 5: 368-390 | 5: 368-391 |
|  |  | 6: 416-437 | 6: 416-437 |
|  |  | 7: 475-495 | 7: 476-496 |
