# Supplementary material for: Identification of a Novel Functional Non-synonymous Single Nucleotide Polymorphism in Frizzled Class Receptor 6 Gene for Involvement in Depressive Symptoms
Source: Front Mol Neurosci. 2022 Jul 7;15:882396. doi: 10.3389/fnmol.2022.882396 (PMC9302575; doi:10.3389/fnmol.2022.882396)
Supplement: Supplementary file 2 [file Data_Sheet_2.DOCX]

***Fzd6*-KI mice identification supplementary file (an example):**

Currently, we have generated a great amount of data on these mice. Below is an example for identification of the pups #213-233.

Firstly, we conducted PCR with two-pair of primers and run gel electrophoresis for the PCR products to screen whether it is wild-type (WT) or mutant mouse.

**
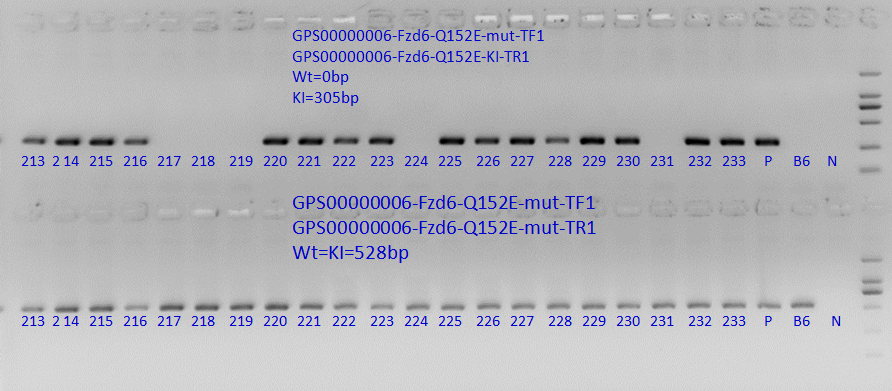
**

Notes:

P: Positive Control

B6: Negative Control, which DNA is from the C57BL/6J mouse

N: Blank Control, no DNA template

Marker: 2000bp\1000bp\750bp\500bp\250bp\100bp

Secondly, we performed Sanger sequencing to validate the gene sequence of each mouse.

**
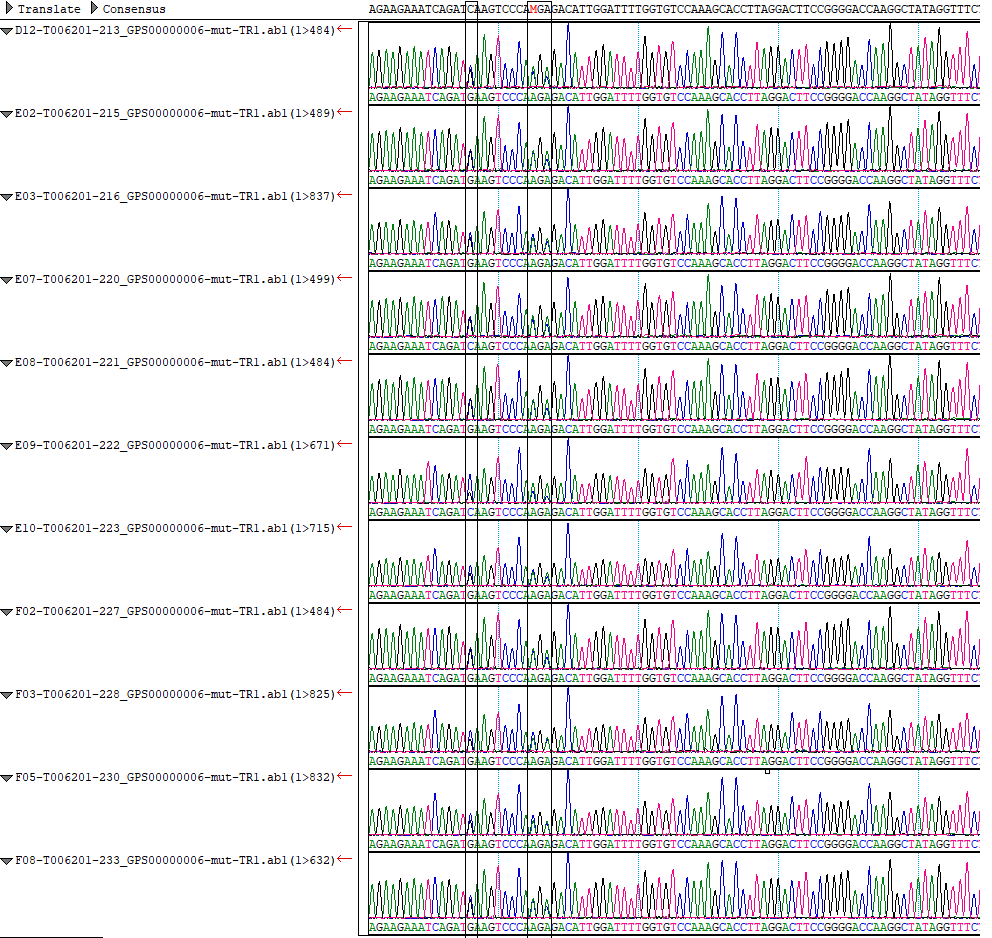
**

**
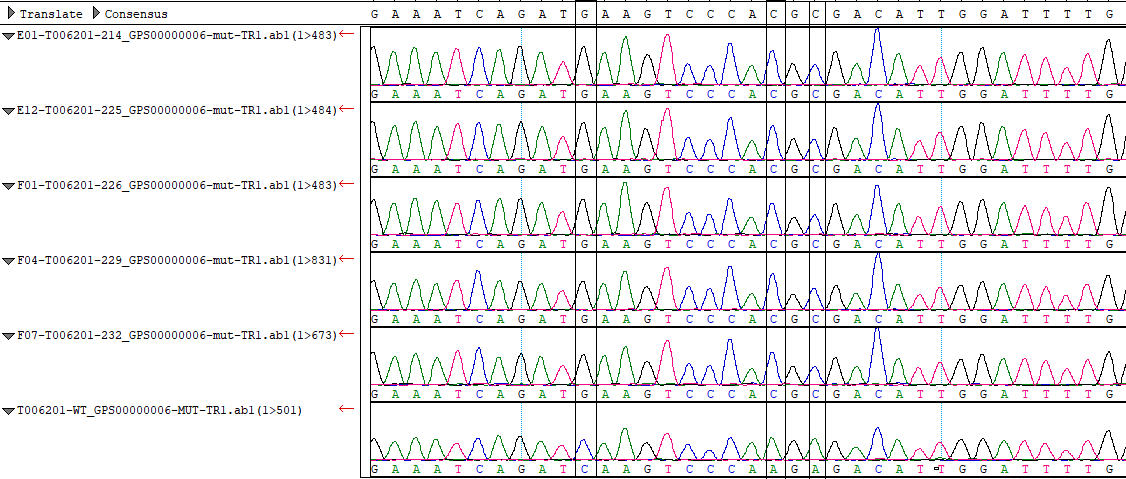
**

Based on above analysis, here is the identification results:

KI/KI (Homozygote mutant mice): 214, 225, 226, 229, 232

KI/WT (Heterozygote mutant mice): 213, 215, 216, 220-223, 227, 228, 230, 233

WT/WT (Homozygote wild-type mice): others

All of the newborn animals were genotyped following the same methods as described above, and only homozygote mice were used for our experiments reported in the paper.
