# Supplementary figures and images for: Identification of a Novel Functional Non-synonymous Single Nucleotide Polymorphism in Frizzled Class Receptor 6 Gene for Involvement in Depressive Symptoms
Source: Front Mol Neurosci. 2022 Jul 7;15:882396. doi: 10.3389/fnmol.2022.882396 (PMC9302575; doi:10.3389/fnmol.2022.882396)

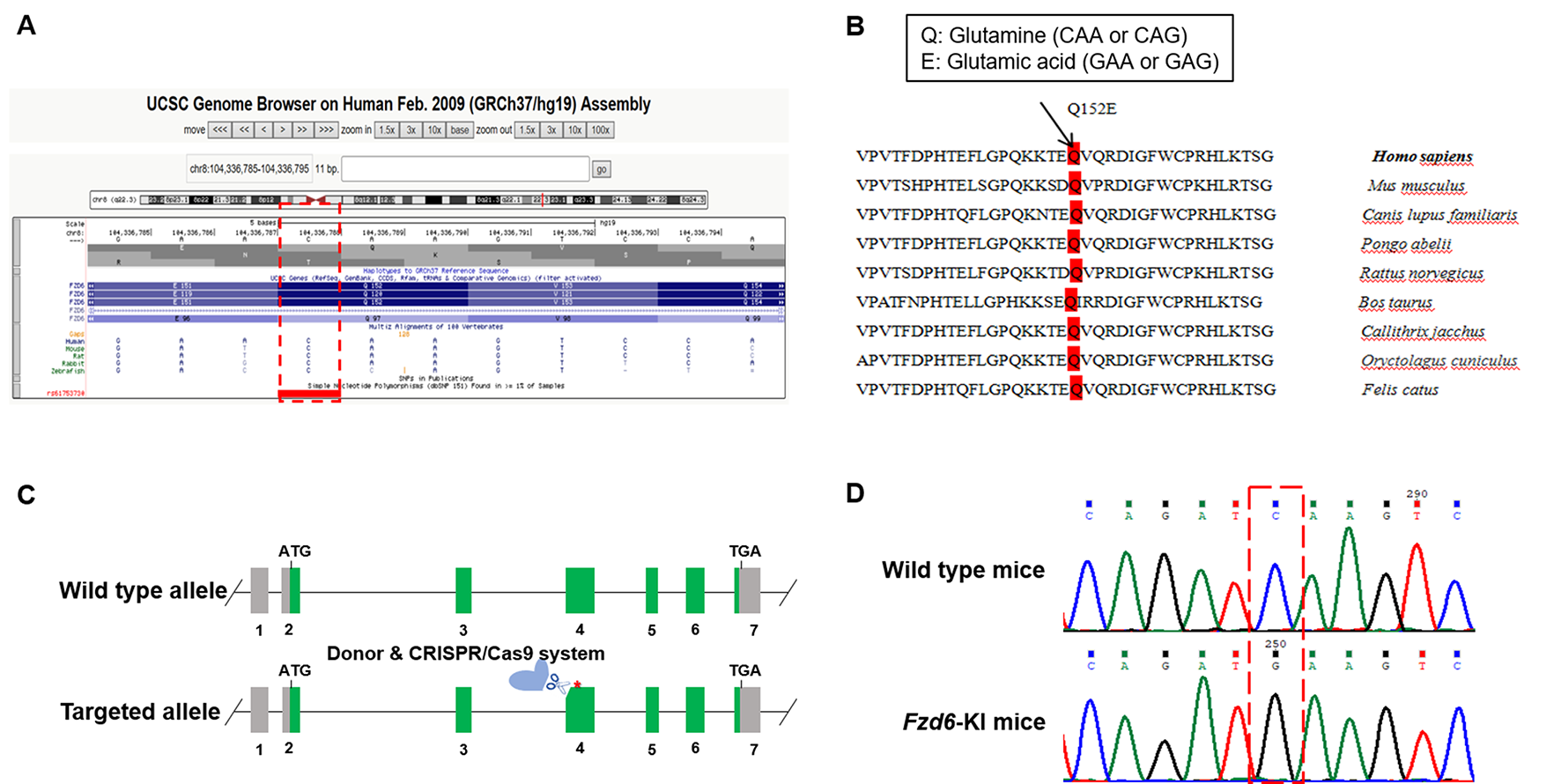

Supplement: Supplementary Figure 1 — Generation of Fzd6-KI mice by CRISPR/Cas9 system. (A,B) Conservation analysis of rs61753730 (Q152E). (A) The UCSC Genome Browser Alignments depict nucleotide alignment of human FZD6 gene and homologs from other species. Position of rs61753730 variant is indicated by dashed red line. (B) Glutamine (Q) is conserved at same point in human, mouse, rat, and other animals. (C) Schematic of strategy for construction of Fzd6 point-mutation mice via the CRISPR/Cas9 system. The rs61753730 is located in fourth exon of Fzd6. Red asterisk indicates mutation site. (D) Sequencing chromatogram to identify WT (with C allele) and Fzd6-KI (with G allele) mice from tail genome DNA. Dashed red line marks location of rs61753730 in two types of mice. [file Image_1.tif]
